# *A20* (*TNFAIP3*) Deletion in Epstein-Barr Virus-Associated Lymphoproliferative Disorders/Lymphomas

**DOI:** 10.1371/journal.pone.0056741

**Published:** 2013-02-13

**Authors:** Midori Ando, Yasuharu Sato, Katsuyoshi Takata, Junko Nomoto, Shigeo Nakamura, Koichi Ohshima, Tamotsu Takeuchi, Yorihisa Orita, Yukio Kobayashi, Tadashi Yoshino

**Affiliations:** 1 Department of Pathology, Okayama University Graduate School of Medicine, Dentistry, and Pharmaceutical Sciences, Okayama, Japan; 2 Hematology Division, National Cancer Center Hospital, Tokyo, Japan; 3 Department of Pathology and Clinical Laboratory, Nagoya University Hospital, Nagoya, Japan; 4 Department of Pathology, Kurume University School of Medicine, Fukuoka, Japan; 5 Department of Immunopathology, Gifu University Graduate School of Medicine, Gifu, Japan; 6 Department of Otolaryngology, Head and Neck Surgery, Okayama University Graduate School of Medicine, Dentistry, and Pharmaceutical Science, Okayama, Japan; The University of North Carolina at Chapel Hill, United States of America

## Abstract

A negative regulator of the nuclear factor (NF)-κB pathway, A20 (TNFAIP3), is inactivated in several types of lymphomas; particularly in diffuse large B-cell lymphoma (DLBCL), classical Hodgkin's lymphoma, and extranodal marginal zone lymphoma of the mucosa-associated lymphoid tissue. These findings suggest that the NF-κB activation is related to A20 inactivation. Recently, A20 inactivation has also been observed in Epstein-Barr virus (EBV)-related lymphomas; however, this occurrence has not been well investigated. Moreover, NF-κB is a key molecule in activated B-cell-like (ABC)-type DLBCL; EBV-associated DLBCL is of the ABC type. Therefore, we focused on *A20* deletions in EBV-associated lymphoproliferative disorders/lymphomas. Using fluorescent *in situ* hybridization analysis, *A20* deletions were identified in 4 of 13 samples from patients with pyothorax-associated lymphoma (PAL) (31%), 3 of 20 samples from nasal-type NK/T cell lymphomas (NKTLs) (15%), 1 of 8 samples of EBV-positive DLBCL of the elderly (DLBCL-e) (13%), but not in any of the 11 samples from individuals with methotrexate-related lymphoproliferative disorder (MTX-LPD) (0%). Among the samples with *A20* deletions, EBV latent membrane protein 1 (LMP-1) expression was detected in all 4 of the PAL samples with *A20* deletions and in the DLBCL-e sample with an *A20* deletion, but not in any of the 3 NKTL samples. This finding indicated that *A20* deletions were not directly related to the EBV latency pattern of lymphomas, although such deletions might be related to the diagnostic category. Immunohistologically, the A20 protein was absent in 2 (15%) of the13 PAL samples, 1 (9%) of 11 MTX-LPD samples, and in none of the 20 NKTL (0%) or 8 DLBCL-e samples. In conclusion, *A20* deletion and/or dysfunctional expression are frequently associated with PALs, and A20 abnormalities may be related to the pathogenesis of PAL.

## Introduction

Nuclear factor (NF)-κB is an important immunological transcription factor affecting cancer development and progression as well as mediating inflammation and autoimmune disease. In malignant lymphomas, the normal NF-κB pathway is dysregulated by many genes and molecular abnormalities, including oncogenic mutations of *MALT1* and *CARD11* [1], [2]. Recently, the inactivation of A20 has been found to play a significant role in the pathogenesis of subsets of several lymphomas [3]–[15].

A20, which is also known as tumor necrosis factor alpha-induced protein 3 (TNFAIP3), negatively regulates the NF-κB activation pathway. A20 is frequently inactivated by deletion, mutation, and/or promoter methylation in several types of lymphomas, such as extranodal marginal zone lymphoma of mucosa-associated lymphoid tissue (MALT lymphoma) [3]–[8], diffuse large B-cell lymphoma (DLBCL) [3], [4], [9], [10], Hodgkin's lymphoma [3], [11], [12], mediastinal large B-cell lymphoma [11], nodal and splenic marginal zone lymphomas [5], [13], follicular lymphoma [3], [4], mantle cell lymphoma [3], [4], Burkitt's lymphoma [4], and AIDS-related lymphoma [14]. Deletion of *A20* has also been reported in NK/T-cell malignancies: NK-cell lymphoma [4]; adult T-cell leukemia [4]; peripheral T-cell lymphoma, not otherwise specified [4]; and Sézary syndrome [15].

In classical Hodgkin's lymphoma (CHL), *A20* alterations are most commonly observed in patients with nodular sclerosis [3], [11]. Schmitz et al. showed that most cases with *A20* alterations were Epstein-Barr virus (EBV)-negative [11]; however, *A20* alterations have been detected in both EBV-negative and EBV-positive patients [11], [12]. Giulino et al. reported *A20* alterations in 6 of 33 patients with AIDS-related lymphoma [14], and that most EBV-positive, AIDS-related lymphoma patients with *A20* alterations did not exhibit latent membrane protein (LMP)-1 expression [14]. As an activation factor of NF-κB, LMP-1 plays an important role in the lymphomagenesis of several types of lymphomas. Giulino et al. suggested that the loss of *A20* may be an alternative mechanism of NF-κB activation in LMP-1-negative, AIDS-related lymphomas [14].

According to previous reports, the constitutive activation of NF-κB seems to be related to the deletion of *A20* in DLBCL, MALT lymphoma, and CHL [3]–[8], [11]. To the best of our knowledge, the association between *A20* deletions and EBV-associated lymphoproliferative disorders/lymphomas has not been well studied. We hypothesized that an association exists, and focused on pyothorax-associated lymphoma (PAL), nasal-type NK/T-cell lymphoma (NKTL), EBV-positive DLBCL of the elderly (DLBCL-e), and B-cell type methotrexate (MTX)-related lymphoproliferative disorder (MTX-LPD). We also investigated the association between *A20* deletions and LMP-1 expression.

## Materials and Methods

### Patient samples

Formalin-fixed, paraffin-embedded samples were obtained from patients with PAL (16), NKTL (33), DLBCL-e (9), and B-cell type MTX-LPD (13) at Okayama University Graduate School of Medicine, Nagoya University Graduate School of Medicine, and Kurume University School of Medicine, Japan. Diagnoses were made using the criteria from the World Health Organization [16]. The inclusion criteria required that each sample consist of more than 80% of the cells being tumor cells and almost all of the tumor cells were positive for EBV-encoded RNA1 (EBER1) in the areas of highest tumor cell density.

EBV status was determined by *in situ* hybridization for EBER1 and immunohistochemical analysis for the presence of LMP-1 and EBV nuclear antigen (EBNA)-2. All samples were obtained with the approval of the Institutional Review Board (IRB) at Okayama University. The samples were limited to excess human material; therefore, the IRB exempted the need for written consent from the patients.

### Immunohistochemical analyses

Detection of A20 and EBV was performed on paraffin sections using the automated Bond Max stainer (Leica Biosystems, Melbourne, Australia). The primary antibody used and the dilution rate were A20 (EPR2663 [1∶100]; Epitomics, Burlingame, CA USA). EBV was detected by *in situ* hybridization for EBER1 (EBER1; Novocastra, Newcastle, UK). In accordance with previous reports, tumors that comprised of at least 20% of A20-positive cells were scored as positive [14]. When the internal positive control cells were not clearly positive for A20, the sample was classified as “undetermined” or “equivocal”. In the “undetermined” groups, the tumor cells were negative, and in the “equivocal” groups, the tumor cells were weakly positive.

Further immunohistochemical testing was performed using the automated Benchmark XT slide stainer (Ventana Medical Systems, Tucson, AZ, USA). The primary antibodies and the dilution rate used were as follows: CD20 (L26; [1∶200]; Novocastra); CD3 epsilon (PS-1; [1∶50]; Novocastra); CD5 (4C7; [1∶100]; Novocastra); CD10 (56C6; [1∶50]; Novocastra); CD56 (IB6; [1∶25]; Novocastra); Ki-67 (MIB-1; [1∶5000]; Novocastra); LMP-1 (CS1-4; [1∶50]; Novocastra); and EBNA-2 (PE2; [1∶20]; Dako, Glostrup, Denmark). Membranous, cytoplasmic, and/or paranuclear dot staining for LMP-1 was evaluated as positive. The percentage of positive tumor cells was categorized as follows: positive (>50%); intermediate (<50%); and negative (0%).

### Fluorescence in situ hybridization

Dual-color fluorescence *in situ* hybridization (FISH) of paraffin sections was performed using the spectrum orange-labeled A20 probe (BAC clones RP11-783B20) and spectrum green-labeled centromeric probe for chromosome 6 (CEP6) (Vysis/Abbott Molecular Laboratories, Des Plaines, IL, USA) following the manufacturers' instructions [17]. The cell was scored only when two internal positive control signals (CEP 6) were present, and the signal ratio of A20 to CEP6 was calculated to evaluate the *A20* status. In DLBCL-e, NKTL, and MTX-LPD samples, the threshold for determining *A20* biallelic deletions was the fraction of signals ranging from 20% to 60%, and that for monoallelic deletions was from 60% to 80%. For PAL samples, each range was set from 20% to 40% and from 40% to 80% for biallelic and monoallelic deletions, respectively. These ranges were different because there were smaller overlaps of cells in the PAL samples, and the non-tumor cell contamination was less than that in other subtypes.

### Statistical analysis

Differences in characteristics between the lymphoma subtypes were determined using the chi-squared test, Fisher's exact test, Student's *t*-test, or Mann–Whitney *U*-test, as appropriate. All data were analyzed with the STATA software (version 10.0; Stata Co., College Station, TX, USA). A *P* value of <−0.05 was considered statistically significant.

## Results

### *A20* deletion by FISH (Table 1)

**Figure 1:**
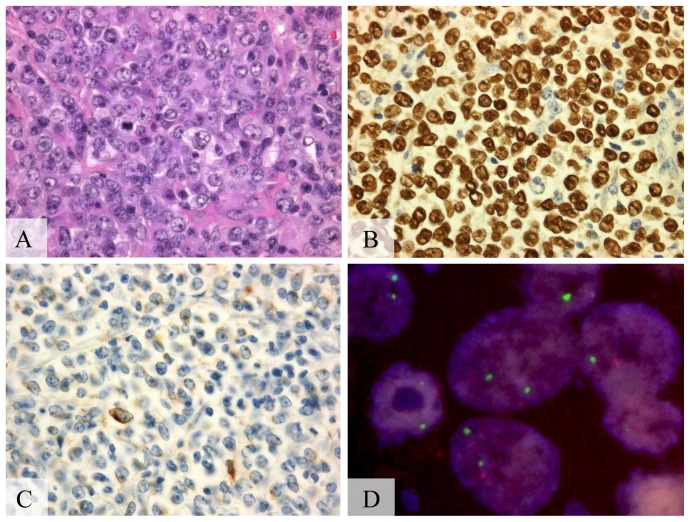
A20 monoallelic deletion in pyothorax-associated lymphoma. (A) Diffuse proliferation of lymphoid cells, (hematoxylin-eosin stain, Olympus BX51, magnification ×200; inset ×400). (B) Positive signals in the nucleus of almost all tumor cells, (Epstein-Barr virus encoded RNA1, Olympus BX51, magnification ×400). (C) Positive signals in >50% of the tumor cells, (latent membrane protein-1, Olympus BX51, magnification ×400). (D) Monoallelic deletion of A20 detected by fluorescent *in situ* hybridization. A20 probe (orange) and chromosome 6 centromeric probe (green) (Olympus IX71, colors corrected after acquisition with Adobe Photoshop).

**Figure 2:**
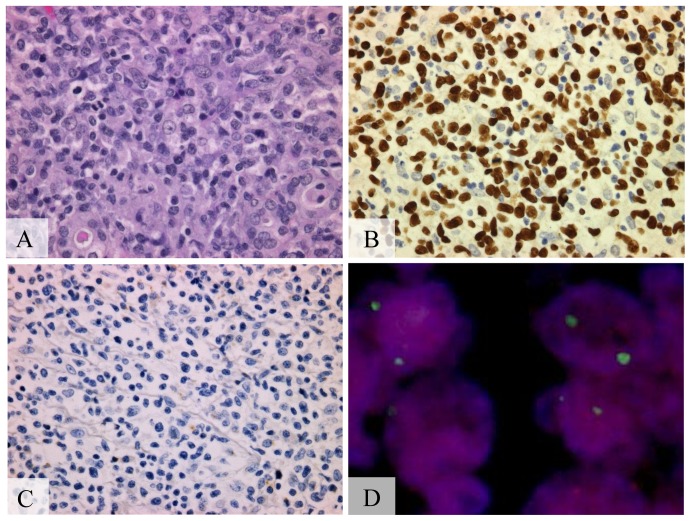
A20 biallelic deletion in nasal-type NK/T cell lymphoma. (A) Medium-sized lymphoid cells with slightly irregular nuclei and mitosis, (hematoxylin-eosin stain, Olympus BX51, magnification ×200; inset ×400). (B) Positive signals in the nucleus of almost all tumor cells, (EBER1, Olympus BX51, magnification ×400). (C) Negative for latent membrane protein-1 (LMP-1) staining (LMP-1, Olympus BX51, magnification ×400). (D) Biallelic deletion of A20 detected by fluorescent *in situ* hybridization. A20 probe (orange) and chromosome 6 centromeric probe (green). (Olympus IX71, colors corrected after acquisition with Adobe Photoshop).

Thirteen of 16 PAL, 20 of 33 NKTL, 8 of 9 DLBCL-e, and 11 of 13 MTX-LPD samples provided interpretable results from the FISH analysis. Of the 13 PAL samples, *A20* deletions were detected in 4 (31%), a biallelic deletion was detected in 1 (8%), and 3 (23%) had monoallelic deletions (Figure 1). In the 20 NKTL samples, FISH indicated biallelic and monoallelic deletions in 1 (5%) and 2 (10%) samples, respectively (Figure 2). In the 8 DLBCL-e samples, there was no biallelic deletion, and only 1 (13%) sample showed a monoallelic deletion. *A20* deletions were not detected in any of the MTX-LPD samples. Of these 4 lymphoma subtypes, PAL had the highest incidence of *A20* deletions. Using a chi-squared test, there were significant differences in the incidence of *A20* deletions between the PAL and MTX-LPD samples (*P* = 0.044). However, significant differences were not observed between PAL and NKTL samples (*P* = 0.28) or between PAL and DLBCL-e samples (*P* = 0.34)

**Table 1:** Mono- and Bi-allelic deletions of *A20* as determined by fluorescent *in situ* hybridization.

	Monoallelic	Biallelic	Total	*P* value (vs. PAL)
**PAL (n = 13)**	3 (23%)	1 (8%)	4 (31%)	**-**
**NKTL (n = 20)**	2 (10%)	1 (5%)	3 (15%)	0.28
**DLBCL-e (n = 8)**	1 (13%)	0 (0%)	1 (13%)	0.34
**MTX-LPD (n = 11)**	0 (0%)	0 (0%)	0 (0%)	0.044

Abbreviations: PAL, pyothorax-associated lymphoma; NKTL, NK/T cell lymphoma, nasal type; DLBCL-e, EBV positive diffuse large B*-*cell lymphoma of the elderly; MTX-LPD, methotrexate-related lymphoproliferative disorders.

### A20 expression by immunohistochemistry

Immunohistochemical staining for A20 was performed on the samples that provided interpretable results from the FISH analysis, except for 2 of the NKTL samples for which there was insufficient sample. The absence of *A20* was shown in 2 (15%) PAL samples, 1 (9%) of MTX-LPD samples, and in none of the NKTL or DLBCL-e samples (Table 2). Two of the 4 PAL samples with *A20* deletions, detected by FISH, were immunohistochemically negative for A20, and the other 2 samples were equivocal. The other samples, without *A20* deletions, were immunohistochemically positive for A20. None of the NKTL samples were immunohistochemically negative for A20; 2 patients could not be assessed because of the absence of sufficient sample material, and 1 had equivocal staining. All DLBCL-e samples were positive for A20, including the sample demonstrating a monoallelic deletion. For MTX-LPD, 1 sample was immunohistochemically negative.

**Table 2:** Samples with *A20* deletions and/or the absence of A20 by immunohistochemistry.

Sample No.	Diagnosis	A20 deletion	A20 expression	EBER1 (ISH)	LMP-1	EBNA-2
1	PAL	Homozygous loss	−	+	+	+
2	PAL	LOH	−	+	+	+
3	PAL	LOH	+/−	+	+	+
4	PAL	LOH	+/−	+	+	+
5	NKTL	Homozygous loss	u.d.	+	−	−
6	NKTL	LOH	u.d.	+	−	−
7	NKTL	LOH	+/−	+	−	−
8	DLBCL-e	LOH	+	+	+	−
9	MTX-LPD	normal	−	+	+	−

Abbreviations: PAL, pyothorax-associated lymphoma; NKTL, NK/T cell lymphoma, nasal type; DLBCL-e, EBV positive diffuse large B*-*cell lymphoma of the elderly; MTX-LPD, methotrexate-related lymphoproliferative disorders; LOH, loss of heterozygosity; EBER1, Epstein-Barr virus encoded RNA1; LMP-1, latent membrane protein-1; EBNA-2, EBV nuclear antigen-2; +, positive (50% or more); p+, intermediate expression (less than 50%); −, negative (0%); +/−, equivocal positive; u.d., undetermined.

### EBV latency state (Table 3)

**Table 3:** Incidence of *A20* deletion and latent membrane protein-1 (LMP-1) status.

LMP-1 Status	PAL	DLBCL-e	NKTL	MTX-LPD
**LMP-1 +**	4/11 (36%)	1/4 (25%)	0/0	0/4 (0%)
**LMP-1 +/−**	0/2 (0%)	0/3 (0%)	0/10 (0%)	0/5 (0%)
**LMP-1 −**	0/0	0/1 (0%)	3/10 (30%)	0/2 (0%)

Abbreviations: PAL, pyothorax-associated lymphoma; NKTL, NK/T cell lymphoma, nasal type; DLBCL-e, EBV positive diffuse large B*-*cell lymphoma of the elderly; MTX-LPD, methotrexate-related lymphoproliferative disorders; LMP-1: +, positive (50% or more); +/−, intermediate expression (less than 50%); −, negative (0%).

The EBV latency patterns were immunohistochemically examined using LMP-1 and EBNA-2. Results of LMP-1 determinations were obtained for all 52 samples and EBNA-2 for 49 samples for which there were insufficient samples. All of the 12 PAL samples in which LMP-1 and EBNA-2 were examined showed latency III (LMP-1^+^, EBNA-2^+^), and 1 sample was not examined for EBNA-2 but was positive for LMP-1. Ten (50%) NKTL samples exhibited intermediate positivity for LMP-1, and all of the NKTL samples were negative for EBNA-2. All 3 NKTL samples with *A20* deletions were negative for LMP-1. In the DLBCL-e samples, latency I (LMP-1^−^, EBNA-2^−^), II (LMP-1^+^, EBNA-2^−^), and III were found in 1, 4, and 3 samples, respectively. One DLBCL-e sample with monoallelic deletion indicated latency II. Of the MTX-LPD samples, 5 (46%) expressed latency II, 4 (36%) expressed latency III, and 2 (18%) expressed latency I. One sample, immunohistochemically negative for A20, expressed latency II.

## Discussion

In malignant lymphomas, A20 inactivation occurs through deletion of the *A20* locus at 6q23, inactivation mutations, and/or methylation of the *A20* promoter. In addition, some studies have suggested an association between A20 inactivation and EBV infection [11], [12], [14]. Schimitz et al. reported that *A20* mutations or deletions are rarely observed in EBV-positive CHL [11]. However, *A20* alteration is seen in certain EBV-related lymphomas [11], [12], [14]. In a previous study, the majority of EBV-positive AIDS-related lymphoma cases with *A20* alterations did not express LMP-1 [14], and the inactivation of A20 has been proposed as an alternative mechanism for NF-κB up-regulation in LMP-1-negative cases [14].

In the current study, 4 of the 13 PAL samples (31%) showed *A20* deletions by FISH, which was higher than that in previous reports studying other types of non-Hodgkin's lymphomas [5], [7], [8], [10], [11], [14] (Table 4). Compagno et al. showed that A20 inactivation was more common in activated B-cell-like (ABC)-type DLBCL than in germinal center B-cell-like (GC)-type DLBCL (24% vs. 2.2%) [9]. According to previous reports [18], [19], the majority of the EBV-positive DLBCL cases have been identified as having the ABC-phenotype; moreover, PAL cases also express the ABC-phenotype [20]. To the best of our knowledge, no study has described the level of NF-κB activation in PAL. However, constitutive NF-κB activation is a common feature of many ABC-type DLBCL cells [21]. As PAL results in the expression of the ABC-phenotype, we suggest that constitutive NF-κB activation may exist in PAL, and *A20* inactivation may contribute to the pathogenesis of this disease.

**Table 4:** Frequency of literature-reported *A20* deletions by fluorescent *in situ* hybridization.

Authors	Subtypes	Cases reported	Monoallelic	Biallelic	Total
**Novak et al. ** [**5**]	EMZL	n = 11	1 (9%)	1 (9%)	2 (18%)
	NMZL	n = 9	1 (11%)	1 (11%)	2 (22%)
	SMZL	n = 12	0 (0%)	0 (0%)	0 (0%)
**Chanudet et al. ** [**7**]	EMZL	n = 161	9 (6%)	3 (2%)	12 (8%)
**Rossi et al. ** [**13**]	SMZL	n = 101	8 (8%)	1 (1%)	9 (9%)
**Bi et al. ** [**8**]	EMZL	n = 105	7 (7%)	2 (2%)	9 (9%)
**Dong et al. ** [**10**]	DLBCL-GI	n = 71	0 (0%)	13 (18%)	13 (18%)
**Giulino et al. ** [**14**]	ARL	n = 33	1 (3%)	5 (15%)	6 (18%)

Abbreviations: EMZL, extranodal marginal zone lymphoma; NMZL, nodal marginal zone lymphoma; SMZL, splenic marginal zone lymphoma; DLBCL-GI, gastrointestinal diffuse large B-cell lymphoma; ARL, AIDS-related lymphoma.

As mentioned above, Giulino et al. reported that the majority of EBV-positive AIDS-related lymphomas, with *A20* alterations, do not express LMP-1 [14]. However, in the present study, *A20* deletions were observed to coexist with LMP-1 expression in PAL and DLBCL-e samples. Most PAL [20], [22] and DLBCL-e [19] tumors are known to be immunohistochemically positive for LMP-1, and NKTLs are negative, or partially positive, for LMP-1 [23]–[25]. The *A20* deletion does not correlate with LMP-1 expression, and it shows a characteristic latency pattern associated with each lymphoma subtype. Therefore, *A20* alterations might not reflect the previously characterized EBV latency pattern of each lymphoma subtype, but they may reflect the diagnostic category.

In NKTL, Karube et al. showed that *PRDM1* and *FOXO3*, rather than *A20*, contribute to its pathogenesis [26]. In the current study, 3 of the 20 NKTL samples showed the deletion of *A20*; this proportion is not as high as that described in previous reports [5], [7], [8],[10]–14. Therefore, we do not consider *A20* to have a significant role in the pathogenesis of NKTL.

DLBCL-e also exhibits the ABC-phenotype as well as prominent activation of NF-κB [19]. The current study investigated whether or not A20 inactivation was found at high frequencies in DLBCL-e. However, only 1 of the 8 DLBCL-e samples showed *A20* deletions. Although this study suggests that A20 may not contribute to the pathogenesis of DLBCL-e, this conclusion is limited by the small sample size. Further research is required to further elucidate the association between A20 and DLBCL-e.

In cases of MTX-LPD, rheumatoid arthritis —a chronic inflammatory disease―is often present; however, the frequency of A20 deletions of MTX-LPD differs from that of PAL. MTX-LPD and PAL are distinct in that MTX-LPD occasionally regresses after the withdrawal of MTX, all PAL patients have poor prognoses. *A20* alterations may not be seen in lymphoproliferative disorders that occasionally show regression. Furthermore, some of the patients from whom the samples in the present study were taken also received anti-TNFα, in addition to MTX. Therefore, the association between the activation of NF-κB and pathogenesis of B-cell type MTX-LPD may not have been present.

Giulino et al. were the first to report immunohistochemical findings for A20; they observed that patients with *A20* mutations and/or monoallelic deletions were frequently positive for A20 [14]. Immunohistochemically, 3 samples from the present work were found to be negative for A20: 2 samples from PAL patients with biallelic and monoallelic deletions, and 1 sample from a MTX-LPD patient without an *A20* deletion. Therefore, we suggest that in cases where the samples are negative for A20 and do not have biallelic deletions, additional alterations of *A20*, such as a mutation and/or promoter methylation, might be present.

In this study, *A20* deletions and/or dysfunctional expressions were frequently found in PAL samples (Table 1), suggesting that A20 inactivation may contribute to its pathogenesis. The coexistence of an *A20* deletion and LMP-1 expression was detected in PAL and DLBCL-e samples, indicating that *A20* deletions and LMP-1 expression are independent characteristics.
